# Congenital mesocolon anomaly, omental band, and Meckel’s diverticulum: a unique case of a small bowel obstruction

**DOI:** 10.1093/jscr/rjaf500

**Published:** 2025-07-16

**Authors:** Binyam Hundito, Nicholas Michael, Matthew D Nealeigh

**Affiliations:** Department of Surgery, Uniformed Services University/Walter Reed National Military Medical Center, 8901 Wisconsin Ave, Bethesda, MD 20814, United States; Department of Surgery, Uniformed Services University/Walter Reed National Military Medical Center, 8901 Wisconsin Ave, Bethesda, MD 20814, United States; Department of Surgery, Uniformed Services University/Walter Reed National Military Medical Center, 8901 Wisconsin Ave, Bethesda, MD 20814, United States

**Keywords:** congenital mesocolon anomalies, internal hernia, omental wrap, bowel obstruction

## Abstract

With advancements in modern imaging, unexpected intraoperative findings are increasingly rare. We present the case of a 35-year-old female with a history of systemic lupus erythematosus and obesity, on home glucagon-like peptide-1 medication for weight loss, who presented with abdominal pain and imaging findings consistent with mesenteric swirling concerning for an internal hernia and a small bowel obstruction. During exploratory laparotomy, a necrotic omental band was discovered encircling the base of the small and large bowel mesentery. In addition, the entire colon was found to be freely mobile, consistent with congenital mesocolon anomaly. An incidental Meckel’s diverticulum was also identified. Fortunately, all bowel was viable. The necrotic omentum and Meckel’s diverticulum were resected, and the patient recovered uneventfully. This case highlights multiple coincidental rare intraoperative findings and discusses our approach to management.

## Introduction

Congenital mesocolon anomalies (CMA), characterized by defective fixation of the colon to the lateral abdominal wall, are rare but documented in the literature [1, 2]. During normal embryological development, after the midgut rotates and retracts into the abdomen during the first trimester, the ascending and descending colon typically fuse to the lateral abdominal wall and are covered anteriorly by the parietal peritoneum [3].

In rare cases, this fusion is incomplete, resulting in persistent mesocolon and abnormal mobility of the cecum, ascending colon, and descending colon. These anomalies predispose patients to complications such as volvulus and internal hernia, which may lead to bowel obstruction, strangulation, or ischemia [4, 5].

Although there are reports of cecal volvulus and intussusception related to CMA, to our knowledge a CMA leading to herniation through a necrotic omental band has not been previously documented. Our case demonstrates unique findings in a patient with concurrent persistent ascending and descending mesocolon (PAM and PDM) and our approach to managing multiple unexpected intraoperative findings in this setting.

## Case presentation

**Figure 1:**
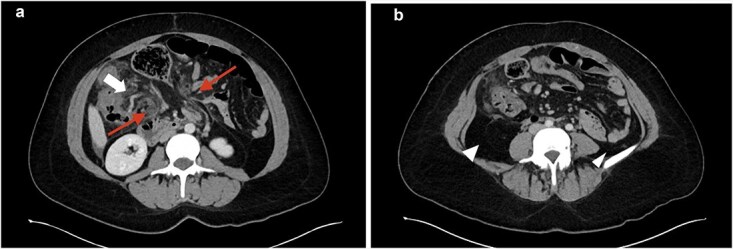
CT images. a: Small and large bowel (top arrow) herniating through omentum (bottom two arrows); b: Intra-abdominal fat displacing colon to the midline (arrowhead).

**Figure 2:**
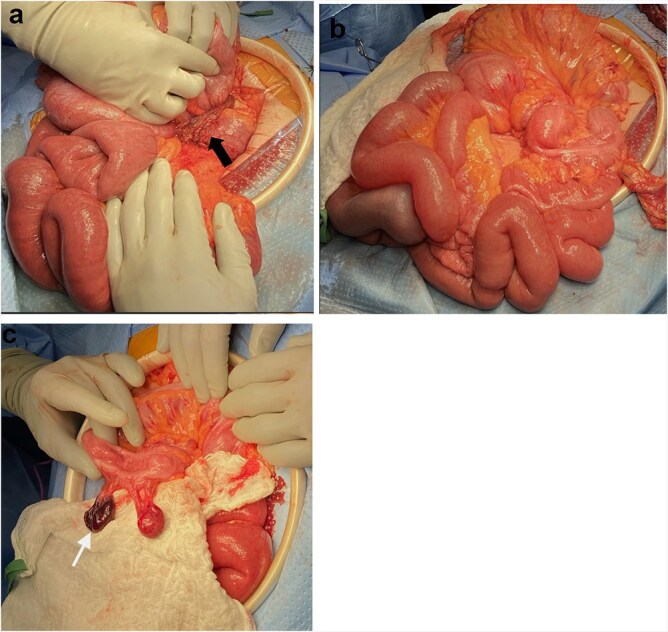
Intraoperative findings. a: Omental band around small bowel (black arrow), with herniation of non-ischemic bowel loops through it. b: Absent lateral attachment of ascending and descending colon. c: Necrotic epiploic appendage (white arrow).

A 35-year-old female presented with acute abdominal pain, nausea, and vomiting. She reported a similar episode one week earlier that resolved without further symptoms. Comorbidities included obesity, lupus, and celiac disease, and she had recently started tirzepitide. Her only prior intra-abdominal surgery was a laparoscopic ovarian dermoid tumor removal. Her abdomen was minimally distended and tender over the left hemiabdomen without peritonitis. Abdominal computed tomography (CT) scan showed distended small and large bowel, mesenteric swirling, pericolonic fat stranding, and medial displacement of both the ascending and descending colon (Fig. 1a and b). With these findings and her ongoing symptoms, urgent exploratory laparotomy was performed. Intraoperatively, the omentum was noted to be thin with multiple defects. The distal omentum was necrotic, wrapped around the base of the mesentery (Fig. 2a and b). All bowel was viable. A Meckel’s diverticulum was identified, measuring approximately 2 cm at the base. A stapled Meckel’s diverticulectomy was performed. Both the ascending and descending colon were freely mobile, lacking lateral attachments. A necrotic epiploic appendage was also resected (Fig. 2c). An appendectomy was also performed. Her postoperative course was uneventful, and she was discharged on postoperative day 3. On pathology, the resected omentum was found to be a hemorrhagic omentum with fibrous adhesions, the Meckel’s diverticulum contained gastric mucosa, and the appendix was normal. At her 4-week follow-up, she continued to recover well.

## Discussion

In this case report, we describe a rare presentation of small bowel obstruction due to an internal hernia through the omentum in a patient with concurrent PAM and PDM. To our knowledge, this is the first reported case of such anomalies occurring together in this manner, highlighting several unique challenges in surgical decision-making encountered during the operation.

Congenital mesocolic anomalies (CMAs), including PAM and PDM, arise from a shared embryological failure of the dorsal mesocolon to fuse with the posterior abdominal wall. However, they differ in clinical presentation, associated complications, and surgical considerations (Table 1). PAM is primarily linked to complications such as cecal volvulus, ascending colon volvulus, intussusception, and internal hernias [2, 5, 6]. PDM, on the other hand, is associated with descending colon volvulus, right-sided sigmoid colon, and vascular anomalies, including radial branching of the inferior mesenteric artery or displaced marginal vessels [1, 2, 7]. In addition to these risks, PDM presents additional challenges in colorectal procedures, as vascular anomalies increase the risk of anastomotic failure when resection is necessary [1]. The simultaneous presence of both PAM and PDM, as seen in this case, is exceedingly rare and compounds the risks associated with altered visceral anatomy. As these are defined by a lack of abdominal attachments, preoperative imaging is generally unable to diagnose these congenital abnormalities. In our patient the CT revealed swirling of the mesentery and pericolonic fat stranding, suggestive of internal herniation, but not specific for any anatomic abnormality. This demonstrates the difficulty in diagnosing CMA preoperatively and the importance of intraoperative recognition and anatomical evaluation to guide appropriate surgical management.

**Table 1:** Comparison of persistent ascending mesocolon (PAM) and persistent descending mesocolon (PDM), highlighting key anatomical differences, complications, diagnostic challenges, and management associated with each anomaly

**Feature**	**Persistent ascending mesocolon (PAM)**	**Persistent descending mesocolon (PDM)**	**Concurrent anomalies (PAM + PDM)**
Definition	Failure of the ascending colon to fuse with the retroperitoneum, leading to increased mobility.	Failure of the descending colon to fuse with the retroperitoneum, resulting in mobility.	Simultaneous lack of retroperitoneal fusion in both ascending and descending colons.
Incidence	Rare; precise incidence not well-documented.	Most common, reported incidence of 2.4% in a cohort of laparoscopic colectomies [1].	Extremely rare; only case reports available
Associated complications	Mobile cecum syndrome, cecal volvulus, cecal bascule, ascending colon volvulus, intussusception and internal hernia.	Volvulus of the descending and sigmoid colon, right-sided descending and sigmoid colon	Combination of complications from PAM and PDM; higher likelihood for internal hernias.
Diagnostic imaging clues	Medial displacement of ascending colon, abnormal position of the cecum.	Medial and caudal displacement of descending colon, right-sided sigmoid colon, radial branching of colic arteries [8].	Medial displacement of both colonic segments, abnormal arterial branching.
Management approach	Laparoscopic/open resection vs fixation (e.g. colopexy) for symptomatic cases. Appendectomy.	Laparoscopic/open resection vs fixation (e.g. colopexy) for symptomatic cases.	Tailored surgical approach based on preoperative imaging and intraoperative findings. Appendectomy.
Intraoperative findings	Mobile cecum, freely mobile ascending colon, abnormal small bowel and other visceral organs position.	Freely mobile descending colon, abnormal small bowel and other visceral organs position.	Entire colon lacks lateral attachments
Reported cases	PAM with herniation through foramen of Winslow, Ascending colon volvulus [5, 6]	Okada *et al.* documented 13 cases of PDM in a laparoscopic colectomy cohort [1].	Concurrent anomalies reported in individual cases; no significant cohorts.
Prognosis	Good with early diagnosis and appropriate surgical management.	Good with early diagnosis and appropriate surgical management.	Potentially more complex due to compounded risks from both PAM and PDM.

Multiple omental defects were also identified and were the ultimate cause of obstruction in this case. Whether these were congenital or related to her CMA is unknown, but the CMA did create the potential for a much worse outcome as they allowed all of the small and large bowel to become involved in the hernia. Omental hernias are exceedingly rare. Wei *et al.* reported a case of small bowel obstruction caused by internal herniation through an omental band in an elderly patient, and suggested the herniation resulted from a congenital abnormality of the omentum rather than a prior surgical insult [9]. While this could have been the case for our patient as well, an alternative possibility is our patient’s recent weight fluctuations. She started Tirzepitde and lost 30 lbs in the months leading up to her presentation, and while not previously described, in theory weight loss like this could have resulted in the development of omental defects and/or omental necrosis.

Additionally, a Meckel’s diverticulum was found during exploration and was managed with a diverticulectomy. This approach aligns with resecting incidentally discovered Meckel’s diverticula in young patients to prevent future complications such as gastrointestinal bleeding, obstruction, and inflammation [10]. An appendectomy was also performed prophylactically to reduce the risk of future diagnostic confusion, particularly in the setting of CMA. This is analogous to the routine appendectomy performed during a Ladd’s procedure for intestinal malrotation, which aims to prevent future misdiagnosis of appendicitis due to atypical positioning of abdominal structures [11].

This case underscores the unique consequences of CMA and our intraoperative decision making guided by this patient’s unique anatomy. Preoperative imaging was concerning due to the mesenteric swirling, and in the setting of her ongoing abdominal pain, it was a straightforward decision to proceed urgently to the operating room. Imaging was not able to predict; however, the ultimate cause of her presentation and the other abnormalities encountered, highlighting the need for adaptability in the operating room.

## Learning points

- Concurrent PAM and PDM are exceedingly rare but may lead to myriad complications and presentations
- CMA are difficult to diagnose on preoperative imaging and are typically only identified once in the operating room
- With no available guidelines, tailored surgical approaches are necessary and prophylactic procedures, such as appendectomy, should be considered to prevent future complications
